# Biosensors as transformative tools for detection of lung cancer based on non-coding RNA

**DOI:** 10.1186/s12935-026-04435-8

**Published:** 2026-08-06

**Authors:** Sina Taghvimi, Zoleikha Asadi, Sajad Ehtiati, Mahzad Nikbakhtrad, Zeinab Chaharlashkar, Fereshteh Rahdan, Seyyed Hossein Khatami, Seyed Mohammad Salehi Behbahani, Hamidreza Zaheri, Ahmad Movahedpour, Hassan Ghasemi, Elahe Soltani Fard

**Affiliations:** 1 Department of Biology, Faculty of Sciences, Shahid Chamran University of Ahvaz, Ahvaz, Iran; 2 Department of Medical Biotechnology, Faculty of Advanced Medical Sciences, Tabriz University of Medical Sciences, Tabriz, Iran; 3 Student Research Committee, Department of Clinical Biochemistry, School of Medicine, Shahid Beheshti University of Medical Sciences, Tehran, Iran; 4 Department of Molecular Medicine, Faculty of Advanced Medical Sciences, Tabriz University of Medical Sciences, Tabriz, Iran; 5 Medical Cellular and Molecular Research Center, Golestan University of Medical Sciences, Gorgan, Iran; 6 Behbahan Faculty of Medical Sciences, Behbahan, Iran; 7 Abadan University of Medical Sciences, Abadan, Iran; 8 Cellular and Molecular Research Center, Yasuj University of Medical Sciences, Yasuj, Iran; 9 Department of Clinical Biochemistry, Research Center for Environmental Contaminants (RCEC), Abadan University of Medical Sciences, Abadan, Iran; 10 Department of Nursing, Malayer School of Medical Sciences, Hamadan University of Medical Sciences, Hamadan, Iran; 11 Student Research Committee, Shahrekord University of Medical Sciences, Shahrekord, Iran; 12 Department of Molecular Medicine, School of Advanced Technologies, Shahrekord University of Medical Sciences, Shahrekord, Iran

**Keywords:** Lung cancer, Non-coding RNAs, Biosensors, Electrochemical, Optical, Electromechanical

## Abstract

Lung cancer remains one of the most prevalent and lethal malignancies worldwide, resulting in approximately 1.5 million deaths annually. Currently, more than 75% of cases are diagnosed at an advanced stage due to the lack of effective early detection methods. Although several conventional diagnostic techniques are available, many exhibits limited sensitivity for early-stage detection and are often associated with high costs and invasiveness. Therefore, there is an urgent need for more accurate, cost-effective, and rapid approaches for early screening. Numerous lung cancer-associated non-coding RNAs (ncRNAs) have been extensively investigated as promising biomarkers for diagnosis, prognosis, and therapeutic monitoring. To leverage these biomarkers, various nanomaterial-enhanced biosensors—utilizing electrochemical, optical, and electromechanical platforms—have been developed for the rapid and selective detection of ncRNAs. This review provides a comprehensive overview of recent advances in nanobiosensor technologies for detecting lung cancer-associated ncRNAs, highlighting their substantial diagnostic potential while critically addressing the technical bottlenecks, reproducibility challenges, and regulatory hurdles that must be overcome for their successful clinical translation.

## Introduction

Lung cancer (LC) remains one of the most common cancers and a leading cause of cancer-related mortality worldwide. For 2026, estimates indicate approximately 229,410 new cases of LC and 124,990 LC-related deaths in the United States alone [1].There are two primary histological classifications of LC: non-small cell lung cancer (NSCLC) and small-cell lung cancer (SCLC), which account for approximately 80–85% and 15–20% of cases, respectively [2]. The primary risk factors for LC include intrinsic characteristics such as sex, genetic susceptibility, and family cancer history, as well as extrinsic factors such as diet, environmental pollution, and tobacco use [3, 4].

Studies on the biology and genetics of LC have highlighted the need for diverse treatment approaches tailored to the specific characteristics of the tumor [5]. LC diagnosis typically involves a variety of techniques, including magnetic resonance imaging (MRI), thoracentesis, computed tomography (CT), positron emission tomography (PET), sputum cytology, and biopsy [6]. However, existing diagnostic procedures are often limited by their invasiveness, high cost, requirement for specialized equipment, and long processing times. Consequently, there is an increasing demand for sensitive, rapid, and user-friendly diagnostic technologies that require minimal instrumentation [7].

ncRNAs, a subclass of RNA molecules, regulate gene expression at the transcriptional and post-transcriptional levels without translating into proteins. NcRNAs serve as key biomarkers involved in critical physiological processes, including cell proliferation [8],differentiation [9], gene expression control, and apoptosis [10]. Over the past few decades, researchers have extensively investigated ncRNAs and their roles in the genesis and progression of LCs. The findings indicate that ncRNAs possess significant potential as diagnostic and therapeutic targets, rendering them promising candidates as cancer biomarkers in clinical evaluation, especially in LCs [11]. Reverse transcriptase quantitative polymerase chain reaction (RT-qPCR) [12], microarray [13], and northern blotting [14] are some of the established methods for ncRNA identification. While these methods possess strong analytical capabilities, they also present notable drawbacks, such as substantial sample volume demands, time-intensive processes, and expensive equipment. Therefore, it is imperative to create novel platforms that can integrate ncRNA screening into conventional point-of-care diagnostic techniques [15]. Recently, researchers have developed numerous biosensor approaches to accurately and sensitively detect cancer biomarkers, specifically ncRNAs. The detection of ncRNAs linked to LC has been facilitated by the expanding application of optical, electromechanical, and electrochemical biosensors. Moreover, the integration of functional nanomaterials in biosensor designs results in improved precision, sensitivity, and selectivity [16–18].

As a result, the purpose of this study is to extensively evaluate ncRNAs as prospective biomarkers for LC diagnosis and prognosis, with a particular emphasis on recent advances in biosensor technology. Herein, we critically examine recent advancements in electromechanical, electrochemical, and optical biosensors, highlighting their translational potential as well as the technical bottlenecks that must be resolved to successfully bridge the gap between laboratory research and routine clinical practice for early and precise LC detection.

## Classification, biogenesis, and functional roles of ncRNAs

**Fig. 1:**
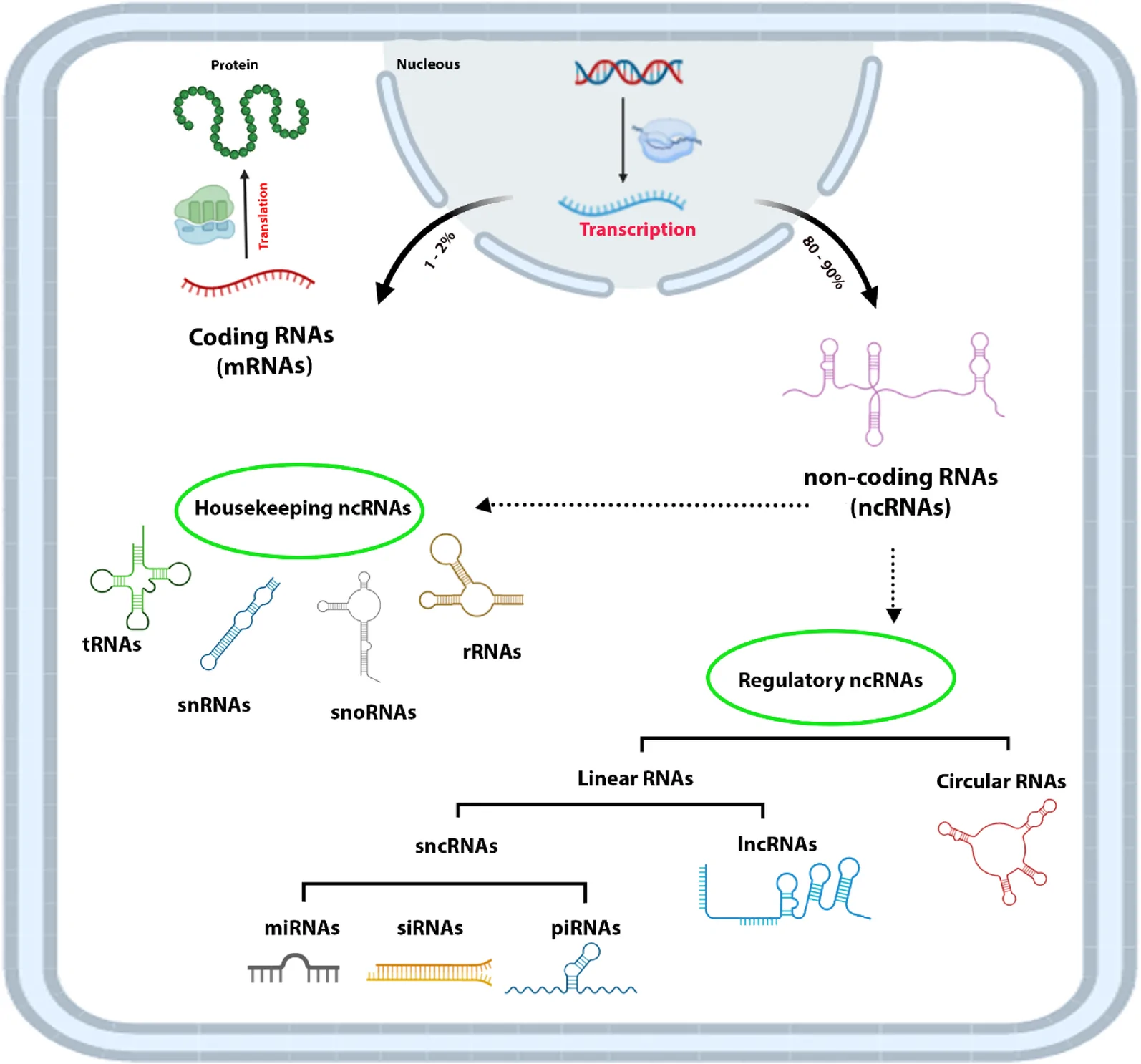
Schematic depiction of ncRNA classifications, their biosynthesis, and the functional effects on target regulation within cellular contexts. ncRNAs are classified into two kinds based on transcript length: small ncRNAs, which are less than 200 bases, and lncRNAs, which exceed 200 bases. MicroRNAs (miRNAs) are produced from diverse genomic areas as either separate transcripts or clusters, first as precursor miRNAs (pre-miRNAs), subsequently evolving into primary miRNAs (pri-miRNAs), and finally maturing into functional miRNAs, which range from 18 to 22 nucleotides, through RNA splicing processes. In contrast, lncRNAs are transcribed from diverse genomic regions using distinct promoters or those associated with separate genes by transcribing the complementary strand or in the opposite orientation of the same strand. The resulting lncRNAs are transformed into circular RNA, tRNA-like, or protein-like structures that can influence various activities, as seen in the picture

The larger portion of the genome that does not code for proteins produces ncRNAs, which regulate gene expression and protein function [19]. Based on molecule size and structure, we separate the ncRNA family into various subgroups. Long non-coding RNA (lncRNA) refers to RNAs longer than 200 nucleotides, whereas small non-coding RNA (sncRNA) refers to any molecule with less than 200 nucleotides [20]. SncRNAs include ribosomal RNAs (rRNAs), small interfering RNAs (siRNAs), tRNA-derived small RNAs (tsRNAs), small nucleolar RNAs (SnoRNAs), microRNAs (miRNAs), and PIWI-interracting RNAs (piRNAs) [21]. There are different types of long noncoding RNAs, such as circular RNAs (circRNAs), pseudogenes, transcribed ultraconserved regions (T-UCRs), and large intergenic ncRNAs (lincRNAs) [22]. (Fig. 1).

### Biogenesis and processing of ribosomal RNA (rRNA)

Ribosome biogenesis is a complicated process that requires precise regulation of rRNAs, mRNAs, and tRNAs transcription. The polycistronic 47 S rRNA precursor is cleaved into smaller rRNAs (18 S, 5.8 S, and 28 S), which are then assembled with ribosomal proteins (RPs) to form ribosomal subunits, and the 40 S and 60 S subunits are transported from the nucleus to the cytoplasm [23]. The process of ribosome formation necessitates the synchronized activities of three distinct forms of RNA polymerase, namely RNA Polymerase I, II, and III. RNA Polymerase I in the nucleolus is responsible for the transcription of the polycistronic 47 S pre-rRNA, which is subsequently cleaved into 18 S, 5.8 S, and 28 S rRNAs [24]. These three rRNAs undergo post-transcriptional modification after interactions with snoRNPs and other protein-processing components. RNA Polymerase III is essential for the transcription of 5 S ribosomal RNA and transfer RNAs in the nucleoplasm. RNA Polymerase II is responsible for the transcription of ribosomal protein-encoding genes in the nucleoplasm [25].

### Structure and mechanism of action of small interfering RNAs (siRNAs)

SiRNA, a synthetic RNA duplex, targets a specific mRNA for destruction. siRNAs consist of two strands: a guide (antisense) strand and a passenger (sense) strand. Together, they form a duplex that ranges in length from 19 to 25 bp and has 3’ dinucleotide overhangs [26]. Dicer cleaves long double-stranded RNA into short duplexes with 3′ two-nucleotide overhangs. Cells are transfected with small double-stranded siRNAs, and the guide strand is inserted into RISC. Perfect complementarity lets this activated protein and nucleic acid pair bind to a single target mRNA sequence. They can then silence a gene by directing cleavage and degradation towards that target sequence [27]. Chemical manipulations of various sections of the siRNA duplex provided valuable insights, revealing that alterations may be readily applied to the 3’-ends whereas the 5’-ends, particularly the antisense strand’s 5’-end, are insensitive to changes [28]. Point mutations have varying consequences based on the place of change. Even with several mismatches, modifications to the sense strand did not influence activity. Single changes near the ends of the antisense strand did not significantly lower activity compared to those put in the center [29].

### Biogenesis and functional roles of tRNA-derived small RNAs (tsRNAs)

Researchers have discovered a separate set of therapeutically relevant small ncRNAs over the last decade. These ncRNAs derived from tRNAs are referred to as tsRNAs [10]. Pre-tRNAs, synthesized by RNA Pol III, undergo modification and processing to develop into functional tRNAs. Based on the cleavage site and whether it occurs in pre-tRNA or mature tRNA, a tRNA can generate several types of tsRNAs [11]. They can be derived from 5′ end, 3′ end, and internal regions [30]. The enzymes involved in the synthesis of tsRNAs are still not completely understood. Nonetheless, several ribonucleases, like Angiogenin, Dicer, and RNase Z, have demonstrated involvement in certain contexts. Argonaute proteins, similar to miRNAs, interact with tsRNAs to regulate mRNA translation by binding to particular 3′ UTRs. Moreover, tsRNAs may exhibit either oncogenic or tumor-suppressive properties [12]. Research continues to uncover the mechanisms underlying their biogenesis and function.

### Small nucleolar RNAs: biogenesis and roles in rRNA processing (SnoRNAs)

SnoRNAs are ncRNAs, 60–170 nt in size. The modification of rRNA conducted by snoRNPs, which are complex structures composed of various components such as snoRNA. These complexes are responsible for sequence-specific 2′-O-methylation [31] and pseudouridylation of rRNA [32]. The nucleolus is the site of post-transcriptional modification to rRNAs, which facilitate rRNA folding and ensure stability. The distinctive sequences of snoRNAs are essential for guiding the assembled snoRNPs to their correct targets [33]. Most snoRNAs reside within introns. After splicing, debranching, and trimming, these RNAs mature and take on two distinct functions. They can either be exported to engage in rRNA processing or remain within the nucleus to contribute to alternative splicing and other yet-to-be-determined activities [34].

### Biogenesis, maturation, and gene silencing mechanism of microRNAs (miRNAs)

MiRNAs are short nc RNAs (approximately 22 nucleotides in length ) that may control a wide range of protein-coding and non-coding genes in animals and plants. This control occurs through post- transcriptional gene silencing, which is enabled by the binding of the miRNA’s 5′ end, commonly known as the ‘seed’, and corresponding sequences in target RNAs [35]. RNA polymerase II transcribes the miRNA gene found in the mammalian genome. Primordial miRNA transcripts (pri-miRNAs) are the molecules formed in the initial phase of miRNA creation and have a hairpin structure [36]. These pri-miRNAs possess a 5’ cap, undergo splicing, and are polyadenylated. The microprocessor complex, consisting of the RNAse III enzyme Drosha and the miRNA-binding protein DGCR8, subsequently processes them [37].

The ~ 60-nucleotide precursor miRNA (pre-miRNA) is transported to the cytoplasm by Exportin 5 and the Ran-GTP complex. Dicer then cleaves the terminal portions of the pre-miRNA, creating a miRNA duplex. This duplex consists of 5′ phosphate groups and has two-nucleotide overhangs at each 3’ end [38]. An Argonaute protein loads one of the guide strands of the miRNA duplex, which aids in the assembly of the RISC. This RISC complex contains the mature 22 nucleotide miRNA. The mature miRNA executes its function by binding to the 3’ untranslated regions (3’ UTR) of mRNAs, thereby promoting either mRNA degradation or translational suppression [39]. In animals, TNRC6 proteins play an important role in the chain of events that leads to translational repression and destruction of target mRNA. TNRC6 proteins impede the action of poly(A)-binding protein (PABP) in protein translation [39]. In this case, the interference changes the connection between the 5′-cap structure and the 3′-poly(A) tail of the mRNA. This mechanism eventually renders the mRNAs available to exoribonucleases [40].

### Biogenesis and function of PIWI-interacting RNAs (piRNAs)

PiRNAs, named after PIWI proteins, are a kind of short non-coding RNA that ranges in length from 21 to 35 nucleotides. Often related to the PIWI subfamily of Argonaute proteins, these specific RNA molecules play a crucial role in repressing transposons during the development of reproductive cells [41]. Species-specific mechanisms synthesize piRNA precursors, while restrictions limit the conservation of piRNA sequences. RNA Pol II transcribes piRNA sequences in mammalian germ cells, producing lengthy, single-stranded precursors [42]. The biogenesis of piRNAs is dependent on two proteins, namely Aub and Ago3 [43]. Aub binds to piRNAs on the antisense strand, causing the cleavage of sense piRNA precursors. This leads to the production of sense piRNAs bound by Ago3. Ago3, on the other hand, binds to piRNAs on the sense strand and cuts antisense piRNA precursors, making antisense piRNAs that connect with Aub [44]. Repeating this cleavage cycle results in the rapid creation of a large number of piRNAs. Secondary piRNA biogenesis, also known as the ping-pong cycle, is the mechanism that creates a vast amount of piRNAs [45]. PiRNAs are responsible for creating suppressive chromatin conditions at target transposons’ promoters, thereby inhibiting their transcription [46]. Alternatively, piRNAs serve as a guide for the PIWI complex to access the transposon mRNA, facilitating the cleavage of the transcript [47].

### Long non-coding RNAs: biogenesis and mechanisms of action (LncRNAs)

Extensive genome transcription leads to the production of numerous lncRNAs. These lncRNAs, longer than 200 nucleotides, do not undergo translation to form functional proteins but are actively engaged in a wide range of biological processes [48]. Similar to miRNAs, lncRNAs are transcribed by Pol II, initiating their biogenesis [49]. RBPs can facilitate the transport of novel lncRNAs to the cytosol after transcription [50]. For lncRNAs with limited exons, nuclear RNA export factor 1 (NXF1) is necessary for efficient export. Once in the cytoplasm, lncRNAs sort into specific organelles and interact with RBPs [51]. Typically, these mature transcripts are distinguished by the presence of 5’ caps and 3’ poly(A) tails [52]. The diverse secondary structures of lncRNAs play a crucial role in facilitating their interactions with DNA, RNA, and proteins [53].

### Biogenesis and regulatory functions of circular RNAs (circRNAs)

Represent an exceptional group of intrinsic ncRNAs, characterized by their identity as RNA molecules that are covalently closed and single-stranded [54]. Although circRNA has been known for many years, it has recently achieved increased attention due to the utilization of advanced deep sequencing and bioinformatics analysis [55].

The process known as back-splicing frequently produces these molecules [56]. This process entails the joining of the 3’ and 5’ ends [57]. They can originate from exons, introns, intergenic regions, UTRs, or even tRNAs [58]. However, our understanding of the precise mechanisms underlying circRNA formation remains incomplete. Similarly, to other regulatory ncRNAs, circRNAs are very important in many biological processes. For example, they help control alternative RNA splicing or transcription [59], and they can also act as competing endogenous RNAs (ceRNAs) or miRNA sponges [60]. CircRNAs undergo transport to the cytoplasm after formation, where they demonstrate their miRNA or protein sponge activities [61].

### Pseudogenes as competing endogenous RNAs in gene regulation

Pseudogenes exhibit sequence similarities to coding genes but have lost their functionality and ability to produce functional proteins. Early stop codons, deletions, insertions, and frameshift mutations prevent them from translating into functional proteins [62]. However, they can regulate their parental genes by creating lncRNAs. Researchers have discovered that the genome expresses thousands of pseudogenes, many of which are specific to specific types of cancer [63]. Pseudogene-derived lncRNAs have emerged as key players in cancer initiation and progression. CeRNAs play a significant role by competitively binding to common miRNAs, hence influencing the expression of both related and unrelated genes [64].

### Transcribed ultraconserved regions (T-UCRs): biogenesis and regulatory functions

T-UCRs are classified as long lncRNAs. Transcription of T-UCRs occurs from 481 highly conserved DNA sequence sections, known as UCRs. In recent years, T-UCRs have been identified and have rapidly emerged as a novel area of investigation [65]. Based on their alignment with established protein-coding genes, scientists categorize T-UCRs, which range in length from 200 to 779 nucleotides, into five groups: intergenic, intronic, exonic, partial exonic, and exon-containing [66]. T-UCRs play an important role in gene expression regulation, including pri-miRNA processing and maturation, mRNA degradation, transcriptional regulation, and epigenetic modification [67].

## LC-Related ncRNAs

The majority of patients with NSCLC already have advanced-stage disease due to the highly aggressive nature of the disease and the difficulties in early diagnosis [68]. The significant role of ncRNA in regulating tumorigenesis is becoming progressively clearer. NcRNAs like miRNA, lncRNA, and circRNA can either enhance or inhibit the progression of LC in affected individuals by modulating vital signaling pathways [69].

The Metastasis Associated in Lung Adenocarcinoma Transcript (MALAT1) is among the initial lncRNAs linked to LC [70]. To investigate its function in LC, Tano et al. suppressed MALAT1 expression in vitro and noticed a decrease in cell motility. This was attributed to a reduction in the activity of genes associated with the reorganization of the extracellular matrix and cytoskeleton [71]. Also, lung tumors have shown a correlation between the upregulation of lncRNA HOTAIR and the occurrence of lymph node metastases. Liu and his colleagues did a lot of in vitro tests where they turned HOTAIR on or off in LC cell lines that had high (SPC-A1, NCI-H1975) or low (A549) levels of HOTAIR expression. They observed that HOTAIR expression was associated with decreased apoptosis and increased cell migration [72].

Numerous studies have demonstrated the association between miRNA regulation disruption and abnormal expression in the development of cancer in different organs, such as LC [73]. Based on their role in carcinogenesis, researchers have recently categorized miRNAs into two groups: onco-miRNAs and tumour suppressor miRNAs. The family members miR-7, miR-31, miR-125, and miR-183 were identified as being disturbed in cases of LC [74].

Recent data suggests that tRNAs have a role in the development of different types of malignancies. In response to hypoxia and other stressful situations, tRNAs can undergo targeted splicing into regulatory pieces. This process enhances LC cell proliferation and affects the cell cycle [75]. Pathogenic alterations in mitochondria-encoded tRNA (mt-tRNA) significantly contribute to the progression of LC by promoting the proliferation of neoplastic cells. Furthermore, there is a close link between tRNA modification and LC [76]. The presence of certain mutations in mt-tRNAs, including tRNA^His^, tRNA^Ala^, tRNA^Leu^, tRNA^Ser^, and tRNA^Thr^, has a significant impact on the development of LC and is extremely likely to be associated with its carcinogenic process [77].

SnoRNAs play a significant role in several physiological and pathological processes. Recent studies reveal that snoRNAs can either inhibit or stimulate tumour growth in certain types of cancer. For instance, NSCLC samples showed increased expression of SnoRNA42, which acted as an oncogene by regulating the expression of p53 [78]. LC stem cells exhibit high expression of SNORA3 and SNORA43, which negatively correlate with survival in individuals with NSCLC. Studies have shown significant dysregulation of SNORA42 in LC stem cells, which makes it a useful marker for identifying these cells [79].

Recent research on the role of piRNA and PIWI in cancer has revealed compelling evidence of a strong association between piRNA/PIWI and different types of malignancies. Peng et al. found a reduction in piR-55,490 expression in LC tissues compared to normal lung tissues. They also observed that blocking piR-55,490 might enhance LC cell proliferation by suppressing mTOR pathway activation in these cells. These findings indicate that piR-55,490 may possess anti-cancer properties in the early stages of LC development [80]. But, when the piR-651 inhibitor was added to LC cells, it was found that piR-651 changed the levels of expression of apoptosis-related proteins caspase-3 and bax, which affected the start and progression of LC [81].

**Fig. 2:**
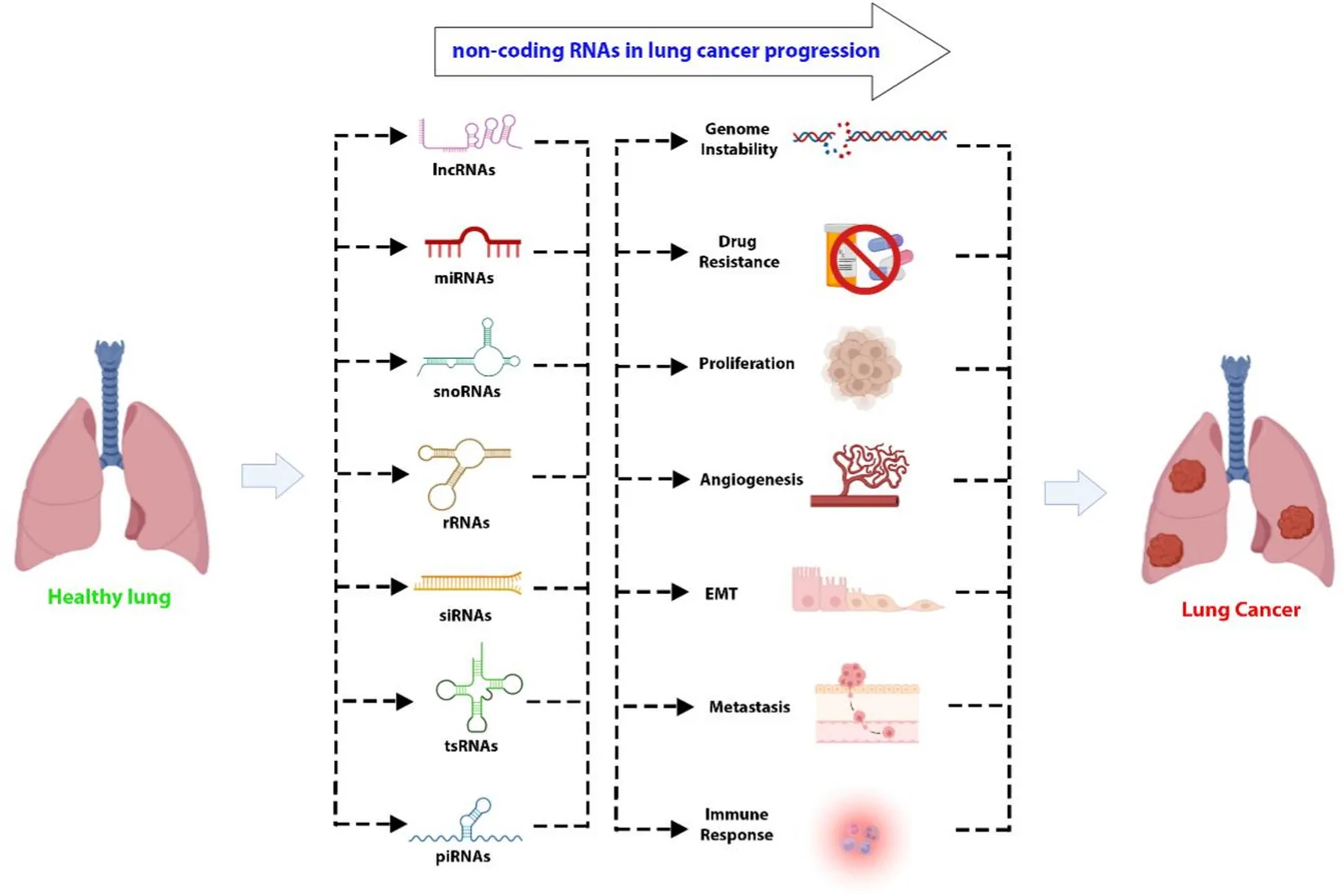
The participation of ncRNAs in lung cancer. ncRNAs are key biomarkers involved in LC progression such as proliferation, cell differentiation, angiogenesis, gene expression control, EMT, metastasis

Research on LC has shown that circRNAs have an inherent regulatory function in the growth and advancement of lung malignancies, and they also hold promise as diagnostic tools. The sex-determining region of the Y chromosome contains *Sry*, a circular transcript with 16 conserved binding sites for miR-138. miR-138 specifically targets H2A histone family member X (H2AX) and controls DNA damage responses in SCLC. A study discovered a significant decrease in the expression of miR-138 in both SCLC tissues and cell lines. Overexpression of miR-138 significantly reduced cell proliferation and induced cell cycle arrest in SCLC cell lines [82]. Hsa_circRNA_103827 exhibited a significant upregulation in LC, whereas hsa_circRNA_000122 showed a notable downregulation. Their expression level has been shown to be correlated with patients’ overall survival. Data confirmed that the expression levels of Hsa_circRNA_404833, hsa_circRNA_406483, hsa_circRNA_006411, hsa_circRNA_401977, and hsa_circRNA_001640 are different in LC tissues [83]. (Fig. 2)

## Biosensors

**Fig. 3:**
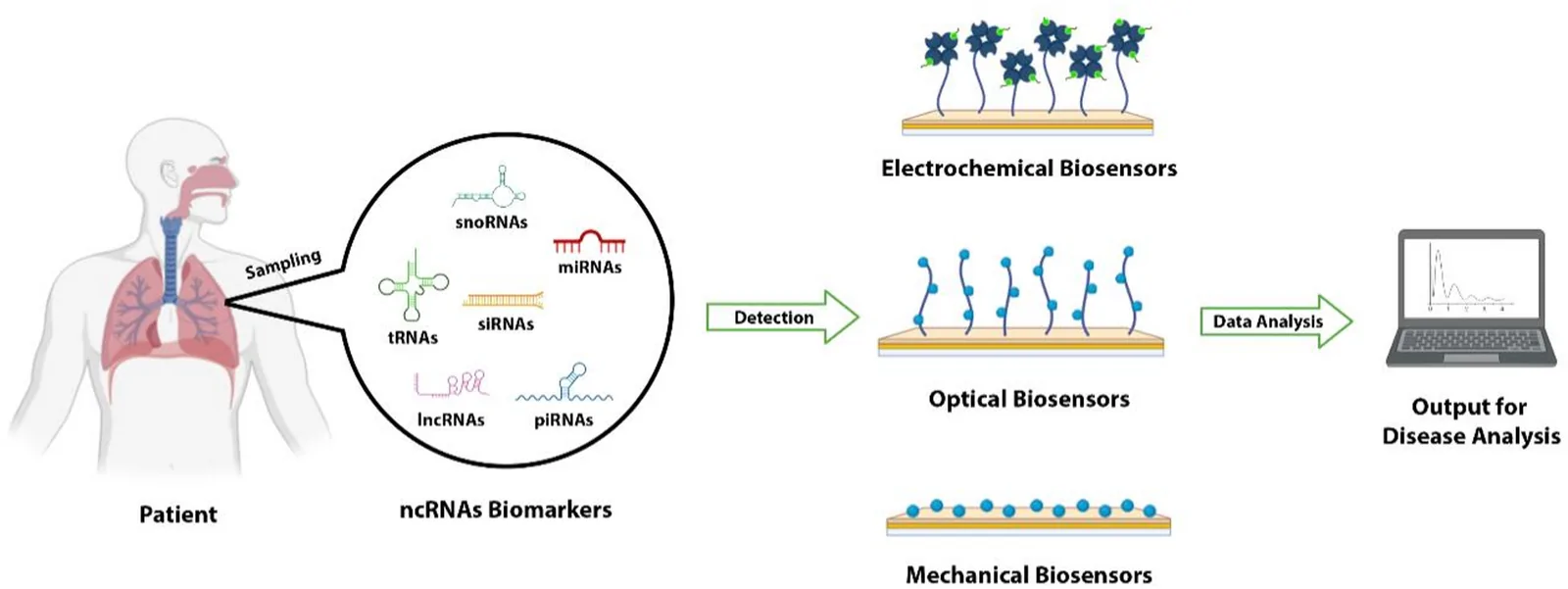
Schematic illustration of biosensor sensing in lung cancer. The detection of ncRNAs linked to LC has been made possible by the widespread use of optical, electromechanical, and electrochemical biosensors

A biosensor is a type of bioanalytical equipment that integrates a molecular diagnostic unit with a physicochemical transducer. Recently, there has been a growing trend towards creating small, highly efficient devices that can operate at the molecular level [84, 85]. Biosensing platforms employ several biorecognition elements, including aptamers, antibodies, nucleic acids, and enzymes. These parts selectively attach to their matching counterparts, like antigens, complementary DNA or RNA, and enzyme substrates [86]. The link between ncRNA biology and biosensor design stems from ncRNAs’ flexibility as regulators in cellular networks, as well as their significance as biomarkers for precise disease detection. ncRNAs (such as miRNAs and lncRNAs) display distinct expression patterns in disorders like cancer, spurring the development of electrochemical, optical, and electromechanical biosensors for their quick and selective detection [87]. These biosensors use nanomaterials to improve sensitivity and selectivity, allowing for early detection of lung cancer, therefore linking ncRNA biology to biosensor design. Furthermore, design concepts for turning naturally existing trans-acting ncRNAs into allosteric RNA molecules that detect chemical signals show modularity between ligand-sensing and target-recognition motifs, enabling new paths for creating ligand-responsive regulatory circuits [88]. Biosensors are classified according to their transduction process, which describes how they transform biological recognition into quantifiable signals. electrochemical, optical, and piezoelectric/acoustic biosensors are the three most often used platforms, with microwave and multimodal systems emerging [89] (Table 1). To ascertain the presence or measure the concentration of the analyzed substance, various sample types can be employed, including solid samples (e.g., tissue) and human biological fluids (e.g., blood, urine, saliva, and cerebrospinal fluid), provided they facilitate the monitoring of the specific biomarker [90]. (Fig. 3). The minimum amount of analyte that can be detected by a biosensor defines its limit range (LR) or sensitivity. In a number of medical and environmental monitoring applications, a biosensor is required to detect analyte concentration of as low as ng/ml or even fg/ml to confirm the presence of traces of analytes in a sample. Despite significant progress in biosensor technology, meaningful clinical translation remains difficult. In addition to excellent analytical sensitivity and specificity, clinically useful biosensors must be reproducible, stable over time, resilient in complex biological samples, simple to use, and cost-effective. Furthermore, large-scale validation studies with diverse patient groups are necessary before regulatory approval and normal clinical deployment may occur. The incorporation of biosensors into portable, wearable, and point-of-care diagnostic devices is a potential development for future healthcare applications. Nonetheless, concerns of standardization, multiplexed detection, manufacturing scalability, and regulatory compliance must be overcome to permit widespread clinical application [91].

**Table 1:** Comparison of different biosensor types used in healthcare diagnostics

Platform	Working principle	Sensitivity	Response Time	Portability	Advantages	Limitations	Typical healthcare applications	References
Electrochemical	Measure current, voltage, or impedance changes due to biochemical interactions	High	Seconds	Excellent	High sensitivity, low cost, easy miniaturization, suitable for POC testing	Electrode fouling, signal drift, interference from complex matrices	Creatinine, glucose, troponins, cystatin C, uric acid	[92, 93]
Optical (SPR/LSPR/Fluorescence)	Detect changes in FL intensity, wavelength, or absorbance	Very High	Seconds–minutes	Moderate	Very high sensitivity, real-time detection, good selectivity	Requires optical components, photobleaching, higher cost	NGAL, cancer biomarkers, viral proteins (COVID-19), DNA/ RNA detection	[94]
Piezoelectric (QCM/SAW)	Detect frequency changes of a piezoelectric crystal due to mass variations caused by biomolecular binding.	Moderate–High	Milliseconds	Good	Simple operation, rapid response, no sophisticated instrumentation	Lower sensitivity, semiquantitative, subjective color interpretation	Glucose, dengue NS1, pH sensing, rapid screening tests	[95]

*CNT* Carbon nanotube, *COVID-19* Coronavirus disease 2019, *DNA* Deoxyribonucleic acid, *MOF* Metal–organic framework, *NGAL* Neutrophil gelatinase associated lipocalin, *NMs* Nanomaterials, *NS1* non-structural protein 1, *pH* potential of hydrogen, *RNA* Ribonucleic acid

### Electrochemical biosensors

An electrochemical sensor is an analytical instrument that employs an electrochemical transducer and a chemically selective layer, known as a recognition element, to rapidly provide accurate information about the target analyte composition [96, 97]. An electrochemical biosensor comprises a biorecognition element that selectively engages with the target analyte, together with an electrochemical transducer that transforms the interaction between the recognition molecule and the analyte into a quantifiable electrochemical signal [98, 99]. This interaction can alter the electrical properties of the biosensor’s interface, such as the speed of electron transfer, its capacity for undergoing redox reactions, and its conductivity. In electrochemical sensing, transducers are essential for producing an electrochemical signal via the interaction between the biorecognition element and the biomarker. This signal can be measured as potential, current, impedance, or conductance [100, 101]. The transducer surface immobilizes recognition components such as antibodies, enzymes, nucleic acid fragments, peptides, and aptamers for the detection of biomarkers. A biorecognition molecule’s specific interaction with a biomarker initiates a biological reaction, which the transducer then detects and transforms. The effectiveness and precision of the biorecognition component have a significant impact on the successful advancement of a biosensor [102].

Electrochemical biosensors have made it possible to accurately find SCLC biomarkers using biofluids that are easy to get to, like serum, urine, and saliva. Recent improvements employ nanomaterials, like graphene and conductive polymers, to improve selectivity and reduce detection thresholds [103]. An ultrasensitive electrochemical biosensor was created via Au NCss-MWCNT-NH2-catalyzed amplification. This biosensor effectively identified spiking lncRNA in clinical serum specimens. The Au NCs/MWCNT-NH2 helped the RNA biosensor work so well because they are very conductive and have a lot of specific surface area. This unique biosensor exhibited an extensive linear range and a minimal detection limit for MALAT1 lncRNA, demonstrating its stability and selectivity [17]. Liu et al. conducted a separate experiment where they created a biosensor that uses electrochemical methods and a 3D DNA origami structure to detect mir-21 in samples from LC patients. They created a biosensor with an upper and a lower component. A ferrocene-labelled DNA molecule with a stem-loop structure at the upper region facilitated binding to the target miRNA. A modified gold electrode surface supported a tetrahedron-shaped DNA molecule containing thiol groups at the lower region. The biosensor demonstrates excellent linearity across various concentrations of microRNA and possesses a low detection limit of 10 pM. These characteristics highlight its potential for clinical use in cancer diagnosis [104]. A novel electrochemical hybrid DNA hydrogel biosensor using a custom-designed DNA probe tagged with ferrocene, was first suggested for the specific detection of miR-21 in LC. The recognition DNA probes were chemically bonded to the DNA strands attached to PAAs to provide a framework for the hybrid DNA hydrogel. The hydrogel biosensor has a detection limit as low as 5 nM (1 pmol) [105]. A novel electrochemical nanogenosensor utilizing a nanohybrid of silver nanoparticles and single-walled carbon nanotubes (AgNPs/SWCNTs) as a label has been created for the detection and analysis of miRNA (miR-25) associated with LC. The nanogenosensor that was created was employed to measure the concentration of miR-25 in two different ranges: 1.0 × 10^− 12^ to 1.0 × 10^− 10^ M and 1.0 × 10^− 10^ to 1.0 × 10^− 8^ M. The limit of detection (LOD) achieved without PCR amplification was 3.13 × 10^− 13^ M. In addition, the nanogenosensor was used to identify miR-25 in human plasma. Utilization of this electrochemical process has significant value in the fields of genetic investigation and early clinical diagnosis [106]. Bettazzi et al. present a technique for detecting miRNA using paramagnetic beads and enzyme amplification in an electrochemical approach. The miR-222 sequence was selected as a model because of its association with LC. Biotinylated DNA capture probes, fixed onto streptavidin-coated paramagnetic beads, form the basis of the suggested bioassay. In order to evaluate the assay, researchers ultimately employed a tiny microfluidic device that allows for the simultaneous analysis of eight different samples. This device has a detection limit of 7 picomoles per liter and a relative standard deviation of 0.15% [107] (Table 2). Electrochemical biosensors have showed great promise for detecting lung cancer biomarkers, notably microRNAs and lncRNAs, with excellent sensitivity and specificity. Advanced materials and signal amplification methodologies have significantly improved their analytical performance, enabling their use in early and non-invasive cancer diagnosis. However, normal clinical deployment still requires greater clinical validation, standardization of manufacturing methods, and the development of portable point-of-care devices.

**Table 2:** Overview of electrochemical biosensors developed for the detection of lung cancer-associated ncRNAs

Biomarker	Method	Signal Enhancer	Samples	LR	Ref
miR-21	colorimetric	AuNPs	Serum	-	[108]
MEG-3	DPV	WS2/DGNFe3O4@C	Serum	1 fM − 100 pM	[109]
miR-21	DPV	AuNPs	-	100 pM − 1 mM	[104]
miR-21	CA, CV, SWV	AuCl_4_	Serum, LC cell line	0.1-3 µM	[110]
miR-21	DPV, CV	ITO	-	10–50 nM	[105]
let-7	SWV	RuO_2_	-	5 fM − 2 pM	[111]
let-7	CV	GOx–DP	LC cell line	10 fM − 10pM	[112]
miR-222	SPEC	-	Calu cell line	-	[107]
MALAT1	SPEC	Au NCs/MWCNT-NH_2_	LC cell line	-	[17]
miR-4732-5p	DPV	GE	Blood	-	[113]
miR-155, miR-21	DPV	AuNP/RGO	-	12–25.7 nM	[114]
miR-155, miR-21	DPV	AuNPs/MoS_2_	-	51.6–59.6 nM	[114]
miR-221	CV	Au disc	LC cell line	-	[115]
miRNA-141	-	GO-CS/PVP-AuNUs	-	-	[116]
miRNA-21	-	GO-CS/PVP-AuNUs	-	-	[116]
miR-21	colorimetric	AuNPs	-	0.0001–1000 pM	[117]
miR-141	SWV	Ag/AgCl	Plasma	200–500 pM	[118]
miR-9	EIS, CV, DPV	Au-NS	Serum	-	[119]
miR-29b-1	CV, SWV	HRP	LC cell line	-	[120]
miR-18a	EIS, CV	AuNPs	Blood	10 fM – 100 pM	[121]
miR-2115-3p	SPEC	AuNPs	Serum	0.1–1000 nM	[122]
miR-155	SPEC	AuNPs	-	-	[123]
miR-155, miR-21, miR-16	-	AuNPs	Serum	1 fM – 10 nM	[124]
miR-21	EIS, CV	Ag/AgCl	Serum	10 aM – 10 pM	[125]
miR-486-5p	DPV	AuNPs	LC cell line	1 fM – 1000 pM	[126]

Au NCs/MWCNT-NH_2_: Gold nanocage coupled with an amidated multi-walled carbon nanotube; AuNPs: Gold nanoparticles; Au-NS: Gold nanostructures; CA: Chronoamperometry; CV: cyclic voltammetry; DPV: Differential pulse voltammetry; EIS: Electrochemical impedance spectroscopy; GE: Gold electrode; GO-CS/PVP-AuNUs: graphene oxide-chitosan@polyvinylpyrrolidone-gold nanourchin; GOX: Glucose oxidase; HRP: Horseradish peroxidas; ITO: Indium tin oxide; SPEC: Screen-printed electrochemical cell; SWV: Square Wave Voltammetry; WS2/DGN: tungsten disulfide/dendritic gold nanostructures

## Optical biosensors

Optical biosensors are characterized by compact dimensions, high sensitivity, and resistance to external fields. Optical biosensors that exploit refractive index variations can differentiate cancerous cells from normal cells [127]. Cancer cells have an unregulated proliferation rate, with protein content significantly exceeding that of normal cells. This results in a measurable difference in refractive index between cancerous and normal cells. The interaction of an incident electromagnetic wave with a normal cell will differ from its contact with a cancer cell. Thus, it will be possible to distinguish normal cells from malignant ones [128]. To develop an optical biosensor for detecting variations in the refractive index, a receptor is necessary to engage with the material being analyzed. This encompasses a range of optical methods, such as evanescent-wave fluorescence, surface-enhanced Raman spectroscopy (SERS), reflectometric interference spectroscopy (RIfS), colorimetric detection, optical coherence tomography (OCT), surface plasmon resonance (SPR), slot waveguides, and metamaterials [127, 128]. Optical biosensors can quickly and accurately find cancer biomarkers like antigens or microRNAs, no matter what recognition mechanism is used. Optical biosensors Primarily utilize gold nanoparticles (AuNPs) as nanomaterials owing to their distinctive characteristics. The color of AuNPs is red, however it can change to purple or blue due to interparticle plasmon interactions. Colorimetric biosensors for miRNA biomarkers can utilize this color shift, discernible to the unaided eye, as a signal output [129].

Several groups have recently investigated the discovery of distinct LC-related ncRNAs using optical biosensors, yielding encouraging results. SPR biosensors have demonstrated significant efficacy in the direct detection of ncRNAs [130]. For example, Calvo-Lozano demonstrated highly sensitive detection of the miR-21-5p biomarker in human plasma samples. They effectively detect and quantify miRNA levels in a single-step experiment, eliminating the necessity for DNA extraction or amplification procedures [131]. Hu et al. developed an innovative technique utilizing SPR sensors for label-free detection of DNA and miRNA-21 at the level of individual nanoparticles. The GNPs were affixed to the ITO glass substrate to constitute the primary component of the sensors. The SPR sensor attained a detection limit of 3 nM at the single nanoparticle level, this SPR sensor detected 3 nM and demonstrated exceptional selectivity for miRNA sequences [132]. A different study by Zhang et al. describes a one-of-a-kind, label-free nanoprobe that is very good at finding miRNA biomarkers. It does this by using LSPR technology on a single DNA-modified gold nanocube (AuNC). This method let us see in real time how the small changes in the LSPR scattering peak location were caused by changes in the dielectric constant while the target miRNA hybridized with ssDNA that had been changed on the surface of AuNCs. There is a 5 pM limit on how much of the AuNC-ssDNA probe can be found in blood samples. This shows that the square shape makes it easier to make nanoprobes that can find very small amounts of LC-related miRNAs [133]. In terms of both qualitative and quantitative detection of miRNAs, the portable SERS sensor demonstrates high sensitivity, specificity, stability, repeatability, and reusability for both qualitative and quantitative miRNA detection. We concurrently analyzed three LC-associated miRNAs (miRNA-21, miRNA-486, and miRNA-375) in buffer and human serum using the SERS sensor array, observing the quenching of the SERS signal in the presence of target miRNA biomarkers. The detection limits for these three miRNAs in human serum were 393 aM, 176 aM, and 144 aM, respectively. The dependable findings indicate that the suggested SERS sensor array may serve as a promising option for the early screening and clinical diagnosis of cancer [134]. Xia et al. demonstrated serum miR-196a concentrations of 20.19 fM in LC patients and 4.98 fM in healthy individuals. Simultaneously, SERS-based detection showed agreement with RT-PCR results.The advanced SERS technique has the capacity for rapid and accurate detection of miRNA expression in cancer patients serum [135] (Table 3).

Optical biosensors, notably SPR-, LSPR-, and SERS-based technologies, have shown great sensitivity and quick detection of lung cancer-related nucleic acid biomarkers, including direct miRNA identification in complicated biological samples. Their superior analytical performance demonstrates their promise for early and non-invasive cancer detection. However, obstacles such as sensor stability, signal repeatability, limited clinical validation, and a lack of standardization must be solved before routine clinical use can be implemented.

**Table 3:** Overview of optical biosensors developed for the detection of lung cancer-associated ncRNAs

Biomarker	Method	Signal Enhancer	Samples	LR	Ref
miR-486	Colorimetric	AgNPs	H460 Cell line	-	[136]
miR-205	LSPR	AuNCs	Serum	10 pM – 1 µM	[133]
let-7	SPR	AuNPs	A549 cell line	200 fM – 2 pM	[137]
miR-205	SERS	AuNPs	A549 cell line	1*10^− 15^ M − 1*10^− 7^ M	[138]
miR-21	Colorimetric	PVDF	Blood	10 nM − 10 mM	[139]
miR-21-5p	Colorimetric	Si_3_N_4_	Blood	0.5–100 nM	[131]
miR-21, miR-486, miR-375	SERS	AgNR	Serum	1 fM – 100 pM	[134]
miR-21	LSPR	GNPs	-	10 nM – 20 µM	[132]
miR-196a	SERS	AgNW/ AuNPs	Serum	1 fM – 100 pM	[135]
miR-205, miR-944	Colorimetric	-	Serum	-	[140]
miR-21	SERS	Core-satellite nanocomposites	Plasma	10 fM – 1 nM	[141]
miR-21	SERS	GNCs	Urine	2.08 pM – 3.31 pM	[142]
miR-196a-5p	SERS	GNCs	Urine	1.77 pM – 2.18 pM	[142]
miR-21, miR-486	SERS	AuNPs	Serum	-	[143]
miR-21	SERS	Au@R6G@AgNP	A549 cell line	12 fM – 18 pM	[144]
miR-125a-5p	SERS	GNCs	A549 cell line	1*10^− 16^ M − 1*10^− 10^ M	
miR-21	SPR	AgNCs	Serum	2 fM – 20 nM	[145]
miR-155	SERS	AgNPs	-	-	[146]
miR-196a-5p, miR-31-5p	SERS	AgNPs	Serum	10 nM – 100 µM	[147]
miR-92a, miR-339-3p	SERS	Au-AgNBs	Serum	-	[148]

AgNB: Silver nanobowl; AgNP: Silver nanoparticle; AgNR: Silver nanorod; AgNW: Silver nanowires; AuNC: Gold nanocube; AuNP: Gold nanoparticle; GNC: Gold nanocage; LSPR: Localized surface plasmon resonance; PVDF: Poly-vinyledene fluoride; SERS: A surface-enhanced raman scattering; SPR: Surface plasmon resonance

### Electromechanical biosensor

Electromechanical systems integrate electrical and mechanical components at the microscale and nanoscale to convert mechanical, chemical, biological, or other sensory responses into electrical signals. Microelectromechanical systems (MEMS) and nanoelectromechanical systems (NEMS) are the two categories into which electromechanical sensors fall [149]. Recent studies have shown that MEMS and NEMS biosensors can be integrated with field-effect transistors (FETs). This is possible because FETs effectively transmit and amplify signals from external sources [150]. A significant feature of these biosensors is their exceptional sensitivity to minute mass changes, as well as their ability to detect molecules without electrical conductivity or optical signals. The principal detection techniques in mechanical biosensors are cantilever, quartz crystal microbalance (QCM), and surface acoustic wave (SAW) (151–153].

Cantilever biosensors transform the molecular recognition of biomolecules into nanomechanical motion, typically associated with an optical readout device. The most commonly used transducer is a clamped rectangular beam, a mechanical construction that is easy to batch-fab at the micrometer scale [154]. A slender quartz disc, with electrodes that serve as sensor surfaces on both sides of the crystal, is the fundamental component of QCM. For QCM to work, the piezoelectric effect must be present. When an electric field is put on quartz from the outside, it puts stress on the crystal’s structure. Applying an alternating voltage to the crystal causes oscillation in a perpendicular direction to the plate’s surface [155]. SAW devices that make horizontally polarized surface shear waves make it easier to make SAW-based biosensors that can find biomolecules directly and without labels in real time. A SAW device usually has a piezoelectric base made of lithium tantalate (LiTaO3) or quartz, with interdigital transducers (IDT) arranged in a flat electrode pattern. The substrate produces SAW by delivering a high-frequency alternating voltage through the IDTs [156].

Park et al. demonstrated a strategic methodology to achieve elevated sensitivity and specificity in miR-21 detection using a mass-sensitive QCM biosensor. This was done using a unique detection method that involved hybrid duplexes formed between miRNA and DNA probes, non-specific intercalation of surface-modified pyrene molecules, followed by amplification of the gold staining signal [157]. A cationic polythiophene derivative has been utilized on a quartz resonator, namely a QCM, for the miR21 assay employing b-PNA and ANP amplification. The proposed method enables the comprehensive capture of all miRNA sequences inside the sample, unlike conventional assays that quantify a singular RNA sequence. miR-21 was successfully detected at clinically pertinent concentrations, with a detection limit of 400 pM [158] (Table 4).

Electromechanical biosensors translate biomolecular interactions into detectable mechanical signals, providing a promising label-free method for identifying lung cancer biomarkers. QCM, MEMS, and NEMS systems have shown good sensitivity and specificity, with signal amplification methods enhancing detection at clinically relevant doses. However, problems such as environmental sensitivity, measurement stability, and insufficient clinical validation persist, emphasizing the need for bigger clinical investigations and the development of robust, portable point-of-care devices.

**Table 4:** Overview of QCM biosensors developed for the detection of lung cancer-associated ncRNAs

Biomarker	Method	Signal Enhancer	Samples	LR	Ref
miR-21	QCM	AuNPs	A549 cell line	0.25 pM – 2.5 µM	[157]
miR-21	QCM	AuNPs	Plasma	1 nM – 10 µM	[158]

QCM: Quartz crystal microbalance; AuNP: Gold nanoparticle

## Conclusions and future perspectives

It is essential to utilize precise and highly sensitive instruments for the early detection of lung cancer, necessitating the identification of specific cancer biomarkers through non-invasive and accurate methodologies to enhance the long-term survival of patients. While ncRNA-based biosensors work well in laboratory settings, their clinical translation necessitates a comprehensive assessment against existing diagnostic criteria as well as the resolution of critical translational challenges. Compared to RT-qPCR—the current gold standard for nucleic acid detection—ncRNA biosensors offer distinct advantages, including faster time-to-result (typically 15–100 min versus 2–4 h), portability for point-of-care deployment, and lower manufacturing costs. However, many reported laboratory platforms still exhibit higher limits of detection (ranging from attomolar to femtomolar levels) compared with RT-qPCR’s typically superior sensitivity [159]. Furthermore, several critical obstacles hinder widespread clinical adoption. These include reproducibility issues resulting from batch-to-batch variability in nanomaterial synthesis, a lack of standardized testing protocols, and stringent regulatory approval pathways (e.g., FDA 510(k)/EUA and CE marking) that require comprehensive performance, safety, and quality manufacturing data. Additionally, generating robust cost-effectiveness evidence to demonstrate clear economic advantages over established methodologies remains a barrier, as multi-site clinical trials require substantial funding and detailed budgeting[160]. Transforming ncRNA biosensors from promising research prototypes into dependable clinical diagnostics will require addressing these issues through multi-center clinical trials directly benchmarking biosensors against RT-qPCR, the development of certified reference materials, early regulatory engagement, and thorough health economic analyses [161]. Consequently, while ncRNAs represent promising biomarkers for early-stage LC, the integration of ncRNA biosensors with advanced nanotechnology holds strong potential rather than immediate readiness to serve as an effective diagnostic tool. The future of electrochemical, optical, and mechanical biosensors for sensing ncRNAs in LC depends heavily on overcoming these scalability barriers. Electrochemical biosensors, with their low cost, could benefit from refined signal amplification to detect trace amounts of LC-specific ncRNAs. Optical biosensors, particularly plasmonic and fluorescence-based techniques, offer real-time monitoring capabilities crucial for validating ncRNA dynamics. Meanwhile, mechanical platforms, such as microcantilevers and piezoelectric systems, provide innovative physical detection mechanisms. Moving forward, bridging the gap between research and clinical application will require rigorous optimization of manufacturing, robust quality control, and the strategic integration of biosensors with emerging technologies like artificial intelligence (AI) and microfluidics to enhance analytical performance and scalability.

## Data Availability

Data sharing is not applicable to this article.
